# Exploratory Mendelian randomization analysis: The genetic susceptibility of CT examination preference and the risk of hypertrophic cardiomyopathy

**DOI:** 10.1097/MD.0000000000048115

**Published:** 2026-03-20

**Authors:** Qiang Dong, Bing Han, Nana Wang

**Affiliations:** a Department of Radiology, Haidian Hospital, Beijing, China; b Department of Testing and Transfusion, No. 984 Hospital of People’s Liberation Army, Beijing, China.

**Keywords:** computed tomography, exploratory analysis, genetic association analysis, genotype data, hypertrophic cardiomyopathy, Mendelian randomization

## Abstract

Hypertrophic cardiomyopathy (HCM) is a common hereditary heart disease, characterized by myocardial hypertrophy, reduced ventricular cavity, and abnormal myocardial fiber structure. Computed tomography (CT) is a commonly used imaging tool in the diagnosis and risk assessment of cardiovascular diseases. This study aims to conduct an exploratory Mendelian randomization (MR) analysis to investigate the genetic association between the propensity to undergo CT (inferred from genetic variations) and the risk of hypertrophic cardiomyopathy (HCM). In this study, a two-sample MR method was employed. Using the genotype data from the MRC-IEU and FinnGen databases, single-nucleotide polymorphisms related to the phenotype “receiving CT scan” were selected. This phenotype was used as a tool variable for the propensity of CT scan. The CT scan propensity based on genetic prediction was regarded as the exposure factor, and HCM was regarded as the outcome. The associations were estimated using inverse-variance weighting, MR-Egger, and weighted median methods. Additionally, multiplicity tests and leave-one-out analysis were conducted to evaluate the robustness of the tool variable and the stability of the estimated values. In the MR analysis, the inverse-variance weighting method indicated a negative correlation (OR = 2.22 × 10^−16^, *P* = .013). However, the MR-Egger and weighted median methods yielded non-significant estimates with wide confidence intervals, reflecting differences in the sensitivity of different analysis methods to potential biases and leading to inconsistent results across methods. The funnel plot and leave-one-out analysis suggested that the set of instrumental variables included in this analysis was statistically robust. This exploratory MR analysis, using a two-sample framework, investigated the genetic relationship between the propensity for undergoing CT scans and the risk of hypertrophic cardiomyopathy. The differences in the estimated values obtained from different analytical methods indicate the inherent complexity involved in using broad non-biological exposure indicators in such studies. These findings are at an early stage and emphasize the importance of using more precise phenotypic definitions in future research to further explore this potential association.

## 1. Introduction

Hypertrophic cardiomyopathy (HCM) is a common hereditary heart disease,^[1]^ typically characterized by myocardial hypertrophy, reduced ventricular chamber size, and abnormal myocardial fiber structure.^[2–4]^ The hallmark of this disease is an increase in left ventricular wall thickness (>15 mm), which cannot be solely explained by the residual left ventricular state, leading to left ventricular hypertrophy^[4]^ HCM can cause pathological changes such as impaired ventricular diastolic function, left ventricular outflow tract obstruction, myocardial oxygen supply-demand imbalance, and arrhythmias^[5,6]^ These changes further result in clinical manifestations, including heart failure, exercise-induced syncope, and severe impacts on patients’ quality of life and prognosis.^[5,7,8]^

The pathogenesis of HCM is complex, with multiple factors, including genetic background, environmental influences, and cardiac load, contributing to the development of myocardial hypertrophy.^[7,9–12]^ Although the genetic basis of HCM has been widely studied, early-onset HCM (such as in children under 12 years old) still presents unique clinical characteristics, including family history, heart failure symptoms, and left ventricular hypertrophy. The symptom burden in this population is related to the age of onset.^[13]^ Despite this, the imaging diagnosis of HCM still faces many challenges, particularly regarding the role of imaging indicators such as CT scans in the occurrence and progression of HCM.

Computed tomography (CT) is a commonly used imaging tool that is widely applied in the diagnosis and risk assessment of cardiovascular diseases.^[14,15]^ Especially in the screening of coronary artery disease, cardiac structural abnormalities, and tumors, CT has demonstrated its important diagnostic value.^[16–18]^ However, direct evidence regarding the nature of the relationship between the phenotypes (i.e., the results obtained through CT scans) and hypertrophic cardiomyopathy (HCM) is still lacking. The association between the propensity of CT scans and HCM remains unclear.

Mendelian randomization (MR) is an analytical method that utilizes genetic variations as instrumental variables to help distinguish potential causal relationships from confounding factors in observational data.^[19]^ This method is based on the principle that genetic alleles are randomly combined at conception, providing a natural experiment. In this study, we employed a 2-sample MR framework, not to directly evaluate the effect of CT imaging, but to explore whether the genetic predisposition reflected by related genetic variations is associated with the risk of HCM when undergoing CT scans. This analysis is a preliminary study on potential shared genetic factors that could be common genetic factors for both CT scan behavior and HCM susceptibility.

## 2. Materials and methods

### 2.1. Study design

The aggregated data used in this study were sourced from published studies that had received institutional review board approval, thus no further approval was required. This was a secondary analysis using publicly available aggregated data from genome-wide association studies (GWAS). No original CT image collection, processing, or analysis was conducted. Therefore, details related to CT equipment, imaging protocols, segmentation software, or machine learning algorithms were not applicable. It was also not possible to obtain individual-level epidemiological data (such as age, gender, BMI) from these aggregated statistics. A 2-sample polygenic analysis method was employed to explore potential genetic associations between the propensity to undergo CT scans and the risk of hypertrophic cardiomyopathy (HCM).

### 2.2. Data sources

#### 2.2.1. Identification of SNPs associated with CT

Genetic indicators for CT scan tendency selection based on summary statistics from GWAS.

We obtained summary statistics from the publicly available MRC-IEU genome-wide association study (GWAS) database to select genetic covariates for the exposure phenotype - namely, “having a CT scan.” This dataset includes 2536 individuals who underwent CT examinations and 460,474 controls. We initially identified 39 independent single-nucleotide polymorphisms (SNPs). To ensure the quality and independence of these covariates, we applied the following screening criteria in sequence: showing a suggestive association at the genomic level (*P* <5 × 10^−5^); excluding loci with linkage disequilibrium with other SNPs (*r*^2^ <0.001, window size = 10000 kb); excluding SNPs with an *F*-statistic <10 to reduce the bias of weak covariates.

#### 2.2.2. Study results: hypertrophic cardiomyopathy

The HCM data were sourced from the FinnGen project (FinnGen), available at https://gwas.mrcieu.ac.uk/datasets/finn-b-I9_HYPERTROCARDMYOP/. This dataset includes 556 HCM cases and 218,236 control participants. The study has received approval from its Institutional Review Board, and all participants provided informed consent in the original study.

### 2.3. Statistical analysis

We conducted a 2-sample MR analysis using the “TwoSampleMR” package (version 0.5.8) in the R software (version 4.2.3). The statistical tests for the main analysis method (IVW method) were corrected using Bonferroni, and a *P*-value <.05 in both tails was considered statistically significant. We used the inverse-variance weighting (IVW) method to estimate the association between CT scan propensity and the risk of hypertrophic cardiomyopathy, and employed the weighted median method and MR-Egger method as sensitivity analyses. The MR-Egger intercept was used to assess the multiplicative effect and to test whether the intercept was statistically different from zero. The data in this article are from public databases and are exempt from ethical review.

## 3. Results

### 3.1. Study design flow

**Figure 1.**
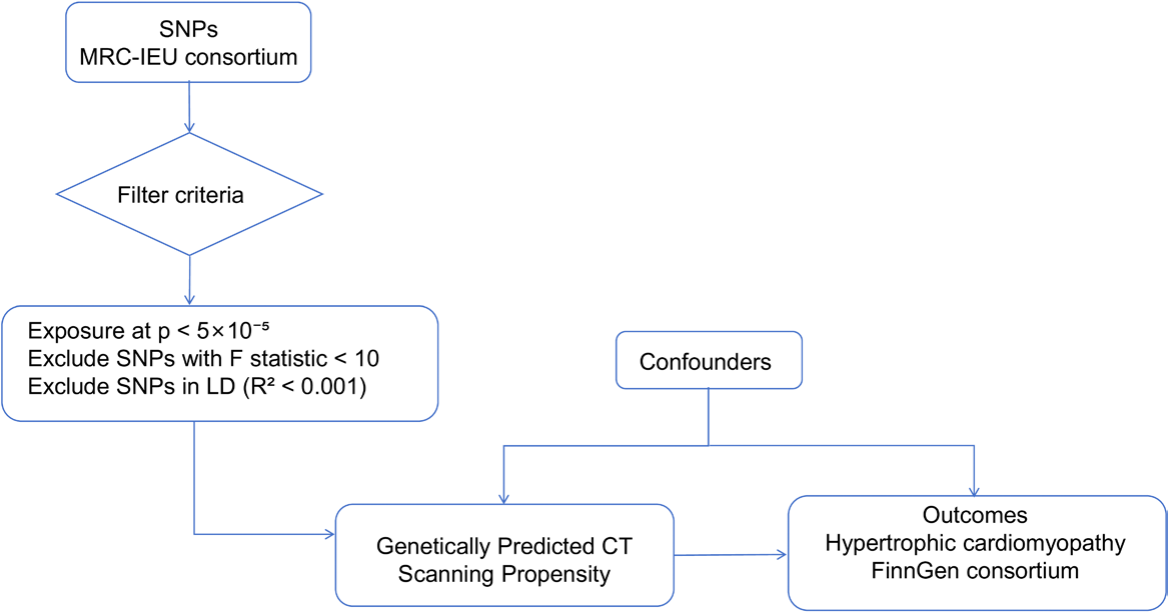
Schematic diagram of the 2-sample MR analysis design. SNPs associated with the phenotype of “having a CT scan” were selected from the MRC-IEU consortium as instrumental variables for the genetically predicted propensity for CT scanning. Their corresponding associations with the outcome, HCM, were estimated using summary statistics from the FinnGen consortium. MR leverages the random assortment of alleles during meiosis to explore potential associations while reducing confounding biases inherent in observational studies. CT = computed tomography, HCM = hypertrophic cardiomyopathy, MR = Mendelian randomization, SNPs = single-nucleotide polymorphisms.

This study adopted a 2-sample MR design, aiming to explore the potential association between genetic predictors of CT scan propensity and the risk of hypertrophic cardiomyopathy. Figure 1 on the left shows the construction process of the exposure instrumental variable: from the pooled data of the GWAS of the MRC-IEU consortium, genetic variants associated with the phenotype “undergoing CT scan” were selected as instrumental variables and were rigorously screened (association significance threshold *P* <5 × 10^−5^, excluding weak instrumental variables with *F*-statistic <10 and SNPs in linkage disequilibrium). These screened genetic instruments were used to predict the core exposure variable, namely the genetic predicted CT scan propensity. Potential unmeasured confounding factors (such as medical accessibility, clinical symptoms, etc) may simultaneously affect the exposure proxy and the outcome. The right side shows the study outcome, namely the data of hypertrophic cardiomyopathy from the FinnGen consortium. This design utilizes the random allocation characteristic of genetic variations to aim to reduce confounding bias in traditional observational studies, thereby conducting exploratory assessment of the association between exposure and outcome.

### 3.2. Genetic covariates for CT scan propensity

**Figure 2.**
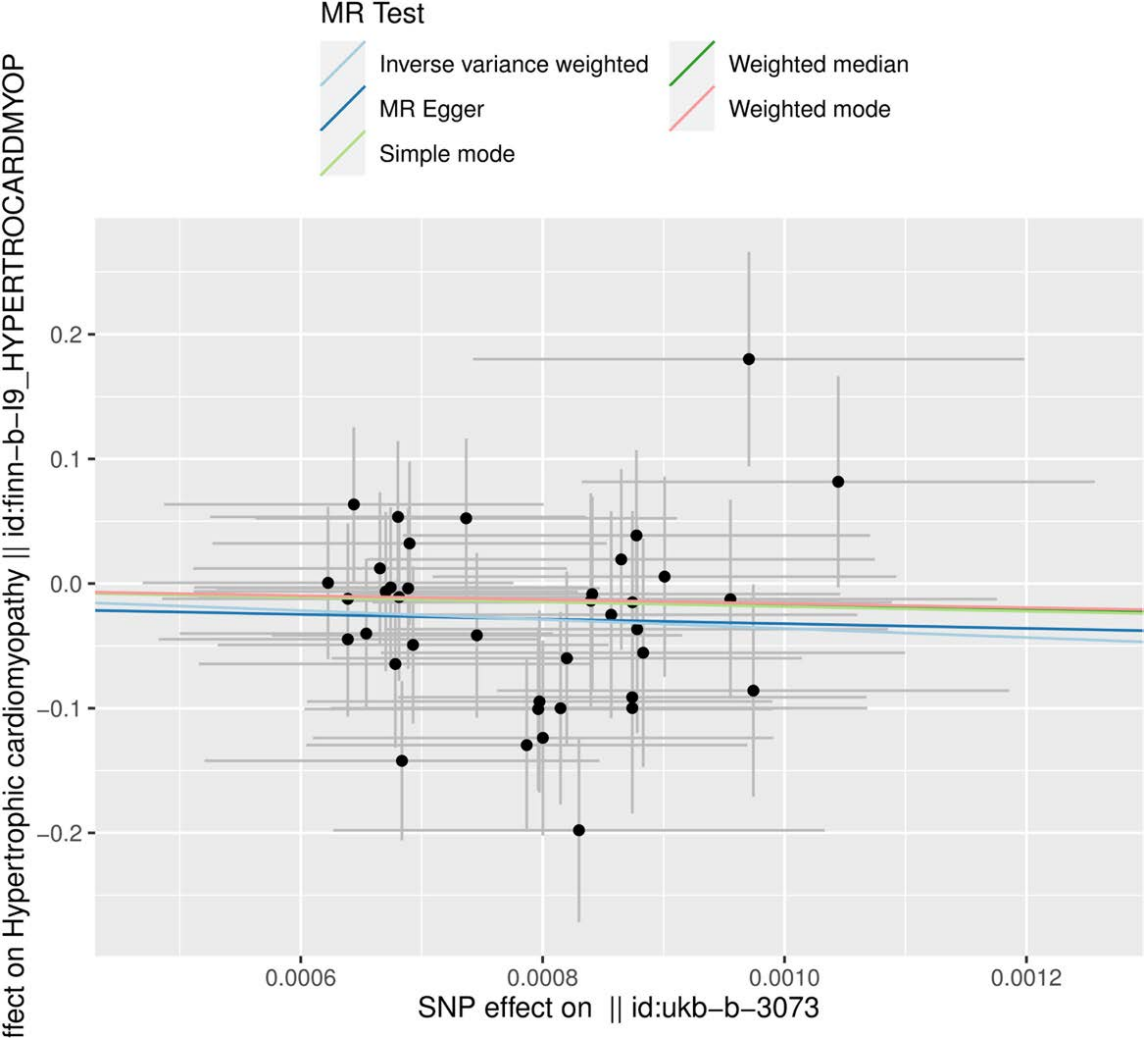
Scatter plot of the genetic associations of the CT scanning propensity with HCM risk. Each point represents an individual SNP. The slope of each line indicates the MR estimate (beta) for the association between the genetically predicted CT scanning propensity and HCM risk, derived from different MR methods. CT = computed tomography, HCM = hypertrophic cardiomyopathy, MR = Mendelian randomization, SNP = single-nucleotide polymorphism.

**Figure 3.**
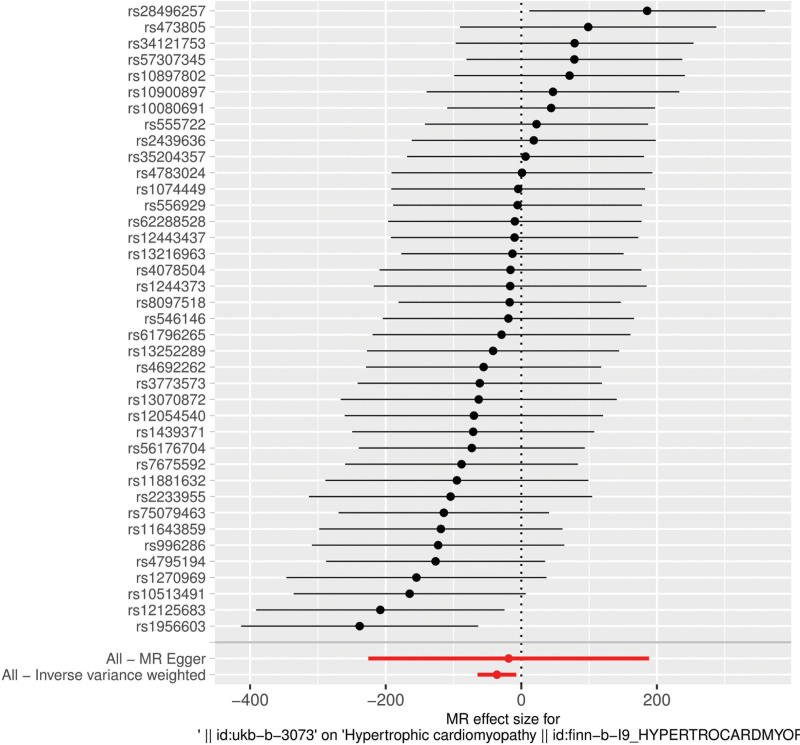
Forest plot of the association between individual instrumental SNPs and HCM risk. The plot shows the effect estimate (odds ratio) and 95% confidence interval for each SNP on HCM risk. HCM = hypertrophic cardiomyopathy, SNPs = single-nucleotide polymorphisms.

We used the genetic prediction of CT scan propensity as an exposure factor and extracted 39 SNPs from it. Table 1 lists all the genetic covariates related to both CT scan propensity and HCM. In Figure 2, each black dot represents a SNP, and its x-axis coordinate represents the effect value of this SNP on the exposure (CT scan propensity), while the y-axis coordinate represents its effect value on the outcome (HCM). The slope of each straight line in the figure represents the association effect suggested by each SNP under different MR methods. Figure 3 shows the association between each genetic variant and the risk of HCM.

**Table 1:** List of genetic instruments for CT and log odds ratios of HCM risk by each instrumental SNPs (GWAS significance with *P* < 5 × 10^−5^ and linkage disequilibrium threshold with *R*^2^ < 0.001).

No.	SNP	Gene	Chr.	EA	OA	EAF.CT	EAF.HCM	CT β (SE)	HCM β (SE)
1.	rs10080691	UST	6	T	C	0.232046	0.2774	−0.000877516 (0.00019319)	−0.0386 (0.0686)
2.	rs10513491	–	3	T	G	0.233854	0.256	0.000786875 (0.000182363)	−0.1296 (0.0687)
3.	rs1074449	GLIS3	9	G	C	0.338425	0.3279	0.000674359 (0.000162683)	−0.0031 (0.0644)
4.	rs10897802	SHANK2	11	A	T	0.280796	0.3389	−0.000736778 (0.000174188)	−0.0524 (0.0639)
5.	rs10900897	–	5	T	A	0.335015	0.296	0.000689963 (0.000163005)	0.0322 (0.0656)
6.	rs11643859	CDH13	16	C	G	0.206518	0.2193	0.000797414 (0.000192313)	−0.0946 (0.073)
7.	rs11881632	–	19	A	T	0.33989	0.2798	0.000678226 (0.000162431)	−0.0645 (0.0671)
8.	rs12054540	SHROOM3-AS1	4	A	G	0.409724	0.3826	0.000638905 (0.000156411)	−0.0447 (0.0621)
9.	rs12125683	–	1	C	T	0.335584	0.3357	−0.000683691 (0.000163175)	0.1422 (0.0639)
10.	rs12443437	UNC13C	15	T	G	0.171383	0.1847	−0.000840907 (0.000204993)	0.0084 (0.0782)
11.	rs1244373	–	14	T	C	0.78605	0.8446	−0.000839744 (0.000206468)	0.0138 (0.0862)
12.	rs1270969	–	9	C	T	0.795446	0.8177	−0.000800229 (0.000190299)	0.1238 (0.0783)
13.	rs13070872	FHIT	3	C	G	0.154411	0.1253	0.000883049 (0.000216618)	−0.0555 (0.0917)
14.	rs13216963	–	6	A	T	0.147111	0.1688	−0.000955205 (0.000220378)	0.0125 (0.0799)
15.	rs13252289	–	8	A	G	0.171462	0.1574	−0.000878096 (0.000207571)	0.0366 (0.0833)
16.	rs1439371	–	4	A	G	0.648547	0.6426	−0.000692862 (0.000161526)	0.0492 (0.0631)
17.	rs1956603	–	14	C	A	0.243861	0.2336	−0.000829961 (0.000203159)	0.1979 (0.074)
18.	rs2233955	C6orf15, PSORS1C1	6	A	G	0.194021	0.2146	−0.000873983 (0.000193603)	0.0912 (0.0931)
19.	rs2439636	–	8	G	A	0.473161	0.4168	0.000665507 (0.000154519)	0.0122 (0.0611)
20.	rs28496257	–	14	G	A	0.138256	0.1488	0.000970509 (0.000227903)	0.1801 (0.0861)
21.	rs34121753	DNAH2	17	G	A	0.577481	0.4532	−0.000680407 (0.000155453)	−0.0535 (0.0609)
22.	rs35204357	–	7	A	T	0.203103	0.1677	0.000900739 (0.000191606)	0.0056 (0.0803)
23.	rs3773573	CACNA2D3	3	A	G	0.455133	0.4921	0.000654133 (0.000154239)	−0.0401 (0.0601)
24.	rs4078504	CTIF, LOC107985147	18	C	A	0.307931	0.2845	0.000681311 (0.0001662)	−0.0109 (0.0671)
25.	rs4692262	–	4	A	C	0.293976	0.2974	−0.000745645 (0.000169469)	0.0415 (0.066)
26.	rs473805	LOC124901342	6	A	G	0.597974	0.6192	0.000643889 (0.000156964)	0.0635 (0.0621)
27.	rs4783024	COTL1	16	C	T	0.495878	0.4384	−0.000622554 (0.000153281)	−0.0006 (0.0611)
28.	rs4795194	ACACA	17	G	A	0.198163	0.2951	0.000796362 (0.000193282)	−0.1007 (0.0656)
29.	rs546146	NOSTRIN	2	G	C	0.471484	0.4601	−0.000638944 (0.000153536)	0.0122 (0.0604)
30.	rs555722	–	13	T	C	0.840772	0.7741	−0.000864904 (0.000209741)	−0.0194 (0.0726)
31.	rs556929	ATP2B2	3	T	C	0.60844	0.671	−0.000688773 (0.000158394)	0.0038 (0.0645)
32.	rs56176704	SLC24A4	14	A	G	0.197496	0.25	0.000820005 (0.00019443)	−0.0599 (0.0697)
33.	rs57307345	ASCL5	1	G	C	0.155591	0.1505	0.00104435 (0.000212193)	0.0817 (0.0848)
34.	rs61796265	LOC105374566	4	G	T	0.172821	0.1586	−0.000856598 (0.000203404)	0.025 (0.0831)
35.	rs62288528	CLNK, LOC105374482	4	C	T	0.377878	0.3421	0.000670258 (0.000158771)	−0.0064 (0.0639)
36.	rs75079463	FENDRR	16	T	G	0.197796	0.2572	0.000874033 (0.000194274)	−0.0999 (0.0692)
37.	rs7675592	–	4	T	G	0.843076	0.8504	0.000974178 (0.000211762)	−0.0859 (0.0853)
38.	rs8097518	MIR4527HG	18	G	A	0.150367	0.2121	−0.000874155 (0.000214574)	0.015 (0.0731)
39.	rs996286	–	12	T	G	0.206258	0.1837	−0.000814848 (0.000190288)	0.1 (0.0774)

Chr = chromosome; CT = computed tomography, EA = effect allele, EAF = effect allele frequency, GWAS = genome-wide association studies, HCM = hypertrophic cardiomyopathy, OA = other allele, SE = standard error, SNPs = single-nucleotide polymorphisms.

### 3.3. Effect analysis of SNP

Figure 2 shows the effect values of each SNP on exposure (CT scan tendency, x-axis) and on the outcome (HCM risk, y-axis). Each point in the figure represents a SNP. Multiple fitted lines represent the overall association slopes obtained from different MR analysis methods (inverse-variance weighted method, MR-Egger method, simple model method, weighted median method, weighted model method). The slopes of each line are close to and negative, suggesting a negative association in the trend of point estimation, but it needs to be combined with other sensitivity analyses for a comprehensive judgment.

Figure 3 shows the effect estimates (β values) and their uncertainties of each independent SNP on HCM risk. The rs numbers of the main SNPs are listed in the figure, and the effect point estimates of most of them are distributed in the negative area (represented by negative bars in the figure), intuitively showing the negative association direction suggested by most instrumental variables. This figure is used to evaluate the heterogeneity of the impact of each genetic instrumental variable on the outcome.

### 3.4. Mendelian randomization analysis of hypertrophic cardiomyopathy

**Figure 4.**
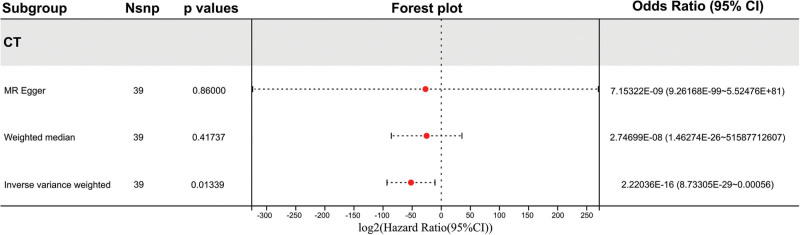
Forest plot of the association between CT scanning propensity and HCM risk using 3 MR methods. The overall effect estimates from the IVW, MR-Egger, and weighted median methods are shown. CI = confidence interval, CT = computed tomography, HCM = hypertrophic cardiomyopathy, IVW = inverse-variance weighted, MR = Mendelian randomization.

We employed IVW, MR-Egger method, and weighted median method to assess the association between the genetic prediction of CT scan propensity and HCM (Fig. 4). The estimated results from different methods were inconsistent: both the weighted median method and the MR-Egger method did not show statistically significant association (weighted median OR = 2.75 × 10^−08^, *P* = .417; MR-Egger OR = 7.15 × 10^−09^, *P* = .860), with wide confidence intervals; while the IVW method suggested a negative association (OR = 2.22 × 10^−16^, *P* = .013). This difference reflects the different sensitivities of the methods to the strength and multiplicity of instrumental variables, so we do not make causal interpretations for any of the results, and merely report them as the observed association pattern in this exploratory analysis.

### 3.5. Multifactorial analysis

**Figure 5.**
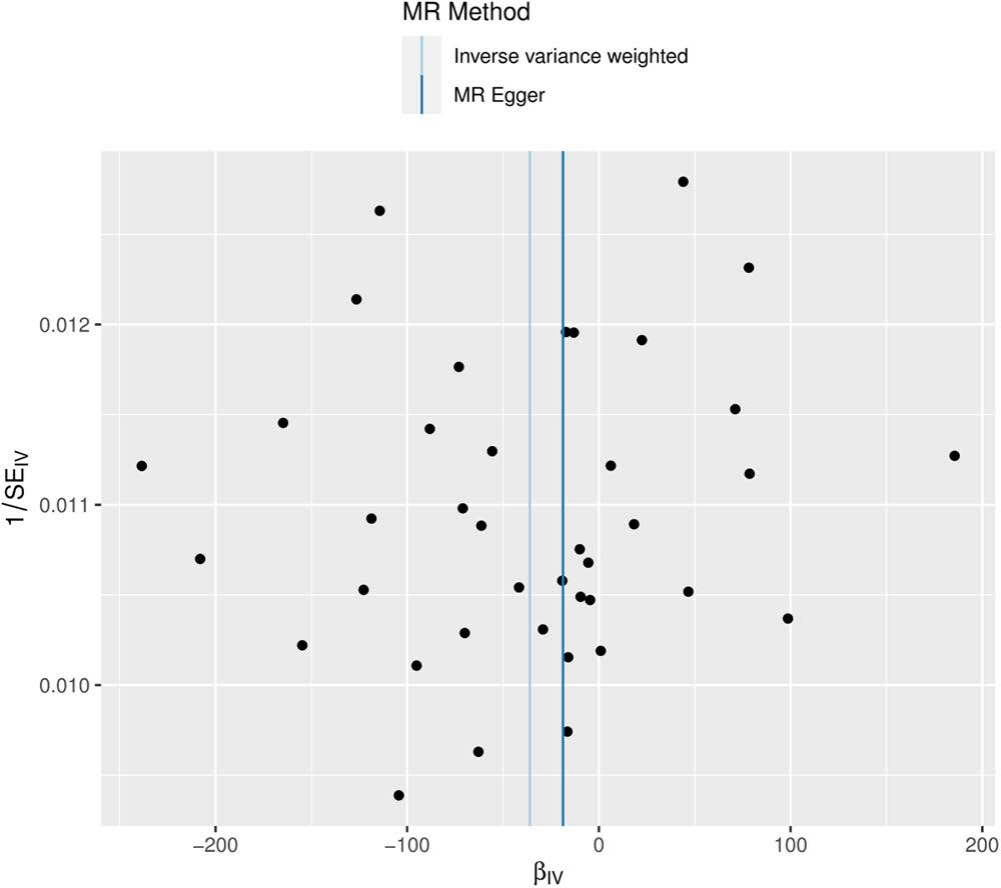
Funnel plot assessing potential directional pleiotropy. The symmetry of the funnel plot is used to visually evaluate the overall heterogeneity and potential directional pleiotropy among the instrumental variable estimates for the effect of CT scanning propensity on HCM risk. CT = computed tomography, HCM = hypertrophic cardiomyopathy, IVW = inverse-variance weighted.

The funnel plot is used to show the relationship between the individual Wald ratio of each SNP and its accuracy. Asymmetry indicates directional multifactoriality. However, it should be noted that when using a small number of funnel plots, it may be difficult to assess the symmetry of the genetic tool (Fig. 5). In this study, the MR-Egger intercept did not show evidence of significant directional multifactoriality (*P* = .86961). These results indicate that there is no significant directional multifactorial effect between CT and HCM.

### 3.6. The impact of individual genetic tools on HCM

**Figure 6.**
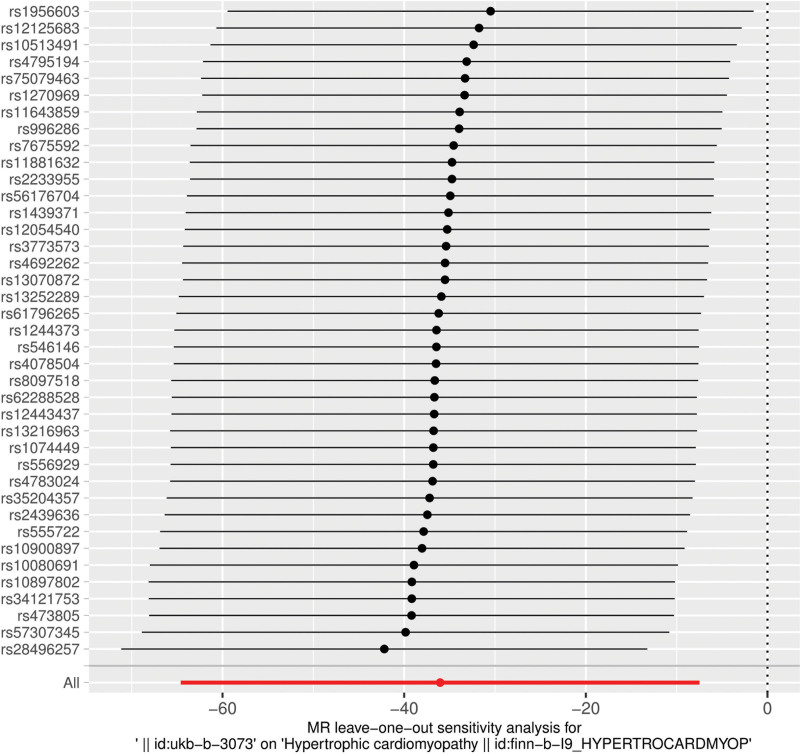
Leave-one-out sensitivity analysis. The plot visualizes the stability of the overall MR estimate for the association between CT scanning propensity and HCM risk by iteratively removing each individual SNP from the analysis. CT = computed tomography, HCM = hypertrophic cardiomyopathy, MR = Mendelian randomization, SNP = single-nucleotide polymorphism.

To verify the influence of each SNP on the overall causal estimate, we conducted a hold-out method analysis. When each SNP was removed one by one and the MR analysis was repeated, there was no significant difference in the estimated causal effect (Fig. 6). Therefore, no single genetic tool significantly affected the estimated effect.

## 4. Discussion

This study employed the MR framework to conduct an exploratory analysis of the association between the genetic prediction of CT scan propensity and the risk of HCM. The key finding was that the estimated results obtained from different MR methods (IVW, MR-Egger, weighted median) were inconsistent.^[20]^ This phenomenon itself is an important discovery, highlighting the methodological challenges encountered when conducting genetic association analyses using such broad and non-biological exposure proxy variables.

The IVW method is a commonly used main approach in MR analysis. In this study, the IVW method indicated that there might be a negative correlation trend between the genetic prediction of CT scan tendency and the risk of HCM.^[21]^ However, this result should be interpreted with caution: Firstly, the corresponding odds ratio (OR = 2.22 × 10^−16^) is extremely small and the confidence interval is extremely wide, which usually indicates weak instrumental variable bias or estimation instability, and should not be directly interpreted as an exact biological effect size; Secondly, neither the MR-Egger method nor the weighted median method showed a significant association. The differences in conclusions from different methods further illustrate that in exploratory analysis, multiple method results should be comprehensively evaluated, and it is not advisable to make inferences based solely on a single method.^[22]^

This study conducted a series of sensitivity analyses to assess the robustness of the results. The MR-Egger intercept test did not reveal any significant evidence of horizontal pleiotropy.^[23]^ The leave-one-out analysis also indicated that a single SNP did not dominate the overall association estimate. Although these findings suggest that the set of instrumental variables has a certain degree of internal consistency statistically, they cannot overcome the fundamental ambiguity and confounding introduced by the very nature of the definition of the exposure variable (i.e., “CT scan propensity”). The symmetry presented in the funnel plot is limited and should be interpreted in the context of a limited number of instrumental variables.^[24]^

This study has several significant limitations. Firstly, and most importantly, there is a problem with the definition of the exposure variable. The “CT” instrumental variable we used represents the genetic predisposition of the behavior of “undergoing CT examination,” which is a composite phenotype that integrates various factors such as medical-seeking behavior, clinical indications, medical accessibility, and potential subclinical conditions that are undiagnosed, rather than a direct biological measurement indicator. The ambiguity of this definition may fundamentally limit effective causal inference.^[25]^ Secondly, we used a threshold with a relatively relaxed genome-wide significance level (*P* <5 × 10^−5^) to obtain a sufficient number of instrumental variables, which may introduce false positive associations.^[26]^ Thirdly, this study is based on publicly available aggregated-level GWAS data, so it is unable to obtain individual-level covariates (such as age, gender, detailed cardiovascular risk factors) for adjustment,^[27]^ nor can it analyze the distribution characteristics of SNPs within case subgroups. Fourthly, even after screening, the average strength of the instrumental variables may still be limited, which can partially explain the extreme OR values and unstable estimation results.^[28]^

In summary, this exploratory MR analysis has completed a systematic exploration of the complex phenotype of “CT scan tendency.” The analysis results indicate that there is inconsistency among different methods, and the effect estimates are unstable. This specifically reveals the methodological challenges and interpretational dilemmas faced in practice when using such broad phenotypes related to behaviors or medical contacts as instrumental variables. Our research process and findings provide important empirical references for future similar analyses and emphasize the necessity of constructing more precise and biologically oriented definitions of exposures (such as quantitative phenotypes derived from imaging).

## 5. Conclusion

In conclusion, this study provides preliminary clues suggesting a potential negative association between CT scan propensity and hypertrophic cardiomyopathy. The observed methodological differences highlight the need for future research to adopt more specific exposure definitions and validated genetic tools in order to further clarify this relationship, rather than directly inferring its clinical application value at this stage.

## Author contributions

**Conceptualization:** Qiang Dong.

**Data curation:** Bing Han, Nana Wang.

**Formal analysis:** Nana Wang.

**Methodology:** Qiang Dong, Nana Wang.

**Writing – original draft:** Bing Han, Nana Wang.

**Writing – review & editing:** Qiang Dong, Nana Wang.

## References

[1] Maron BJ. Clinical course and management of hypertrophic cardiomyopathy. N Engl J Med. 2018;379:655–68. 30110588 10.1056/NEJMra1710575

[2] Geske JB Ommen SR Gersh BJ. Hypertrophic cardiomyopathy: clinical update. JACC Heart Fail. 2018;6:364–75. 29655825 10.1016/j.jchf.2018.02.010

[3] Lioncino M Monda E Verrillo F. Hypertrophic cardiomyopathy in RASopathies: diagnosis, clinical characteristics, prognostic implications, and management. Heart Fail Clin. 2022;18:19–29. 34776080 10.1016/j.hfc.2021.07.004 PMC9674037

[4] Crea F. Acute and chronic heart failure: exciting therapeutic perspectives. Eur Heart J. 2023;44:1–4. 36586417 10.1093/eurheartj/ehac767

[5] Marian AJ Braunwald E. Hypertrophic cardiomyopathy: genetics, pathogenesis, clinical manifestations, diagnosis, and therapy. Circ Res. 2017;121:749–70. 28912181 10.1161/CIRCRESAHA.117.311059 PMC5654557

[6] Maron BJ Rowin EJ Udelson JE Maron MS. Clinical spectrum and management of heart failure in hypertrophic cardiomyopathy. JACC Heart Fail. 2018;6:353–63. 29655822 10.1016/j.jchf.2017.09.011

[7] Ommen SR Mital S Burke MA. 2020 AHA/ACC guideline for the diagnosis and treatment of patients with hypertrophic cardiomyopathy: a report of the American College of Cardiology/American Heart Association Joint Committee on Clinical Practice Guidelines. Circulation. 2020;142:e558–631. 33215931 10.1161/CIR.0000000000000937

[8] Mascia G Crotti L Groppelli A. Syncope in hypertrophic cardiomyopathy (part I): an updated systematic review and meta-analysis. Int J Cardiol. 2022;357:88–94. 35304190 10.1016/j.ijcard.2022.03.028

[9] Ho CY Day SM Ashley EA. Genotype and lifetime burden of disease in hypertrophic cardiomyopathy: insights from the Sarcomeric Human Cardiomyopathy Registry (SHaRe). Circulation. 2018;138:1387–98. 30297972 10.1161/CIRCULATIONAHA.117.033200 PMC6170149

[10] Marian AJ. Molecular genetic basis of hypertrophic cardiomyopathy. Circ Res. 2021;128:1533–53. 33983830 10.1161/CIRCRESAHA.121.318346 PMC8127615

[11] Glavaški M Velicki L Vučinić N. Hypertrophic cardiomyopathy: genetic foundations, outcomes, interconnections, and their modifiers. Medicina (Kaunas). 2023;59:1424. 37629714 10.3390/medicina59081424 PMC10456451

[12] Ingles J Goldstein J Thaxton C. Evaluating the clinical validity of hypertrophic cardiomyopathy genes. Circ Genom Precis Med. 2019;12:e002460. 30681346 10.1161/CIRCGEN.119.002460 PMC6410971

[13] Teekakirikul P Zhu W Huang HC Fung E. Hypertrophic cardiomyopathy: an overview of genetics and management. Biomolecules. 2019;9:878. 31888115 10.3390/biom9120878 PMC6995589

[14] Norrish G Cleary A Field E. Clinical features and natural history of preadolescent nonsyndromic hypertrophic cardiomyopathy. J Am Coll Cardiol. 2022;79:1986–97. 35589160 10.1016/j.jacc.2022.03.347 PMC9125690

[15] Jeon CH Kim YK Chun EJ. Coronary artery vasculitis: assessment with cardiac multi-detector computed tomography. Int J Cardiovasc Imaging. 2015;31(Suppl 1):59–67. 25841665 10.1007/s10554-015-0652-8

[16] Foldyna B Mayrhofer T Lu MT. Prognostic value of CT-derived coronary artery disease characteristics varies by ASCVD risk: insights from the PROMISE trial. Eur Radiol. 2023;33:4657–67. 36719496 10.1007/s00330-023-09430-5 PMC10765563

[17] Košuta D Jug B Fras Z. Prognostic impact of nonobstructive coronary artery disease detected by coronary computed tomographic angiography. Angiology. 2021;72:749–53. 33739163 10.1177/0003319721999494 PMC8326899

[18] Shimabukuro M Saito T Higa T Nakamura K Masuzaki H Sata M; Fukuoka diabetologists group. Risk stratification of coronary artery disease in asymptomatic diabetic subjects using multidetector computed tomography. Circ J. 2015;79:2422–9. 26399764 10.1253/circj.CJ-15-0325

[19] Yu M Harper AR Aguirre M. Genetic determinants of the interventricular septum are linked to ventricular septal defects and hypertrophic cardiomyopathy. Circ Genom Precis Med. 2023;16:207–15. 37017090 10.1161/CIRCGEN.122.003708 PMC10293084

[20] Bowden J Smith GD Haycock PC Burgess S. Consistent estimation in Mendelian Randomization with some invalid instruments using a weighted median estimator. Genet Epidemiol. 2016;40:304–14. 27061298 10.1002/gepi.21965 PMC4849733

[21] Burgess S Thompson SG. Interpreting findings from Mendelian Randomization using the MR-Egger method. Eur J Epidemiol. 2017;32:377–89. 28527048 10.1007/s10654-017-0255-x PMC5506233

[22] Sanderson E Smith GD Windmeijer F Bowden J. An examination of multivariable Mendelian Randomization in the single-sample and two-sample summary data settings. Int J Epidemiol. 2019;48:713–27. 30535378 10.1093/ije/dyy262 PMC6734942

[23] Bowden J Del Greco M F Minelli C Smith GD Sheehan NA Thompson JR. Assessing the suitability of summary data for two-sample Mendelian Randomization analyses using MR-Egger regression: the role of the I2 statistic. Int J Epidemiol. 2016;45:1961–74. 27616674 10.1093/ije/dyw220 PMC5446088

[24] Bowden J Spiller W Del Greco M F. Improving the visualization, interpretation and analysis of two-sample summary data Mendelian Randomization via the radial plot and Radial regression. Int J Epidemiol. 2018;47:1264–78. 29961852 10.1093/ije/dyy101 PMC6124632

[25] Davey Smith G Hemani G. Mendelian randomization: genetic anchors for causal inference in epidemiological studies. Hum Mol Genet. 2014;23:R89–98. 25064373 10.1093/hmg/ddu328 PMC4170722

[26] Burgess S Smith GD Davies NM. Guidelines for performing Mendelian Randomization investigations: update for summer 2023. Wellcome Open Res. 2019;4:186. 32760811 10.12688/wellcomeopenres.15555.1 PMC7384151

[27] Hartwig FP Davies NM Hemani G Smith GD. Two-sample Mendelian randomization: avoiding the downsides of a powerful, widely applicable but potentially fallible technique. Int J Epidemiol. 2016;45:1717–26. 28338968 10.1093/ije/dyx028 PMC5722032

[28] Burgess S Davies NM Thompson SG. Bias due to participant overlap in two-sample mendelian randomization. Genet Epidemiol. 2016;40:597–608. 27625185 10.1002/gepi.21998 PMC5082560

